# Sample descriptors linked to metagenomic sequencing data from human and animal enteric samples from Vietnam

**DOI:** 10.1038/s41597-019-0215-2

**Published:** 2019-10-15

**Authors:** Mark Woolhouse, Jordan Ashworth, Carlijn Bogaardt, Ngo Tri Tue, Steve Baker, Guy Thwaites, Tran My Phuc

**Affiliations:** 1 Usher Institute, University of Edinburgh, Edinburgh, UK; 2 Oxford University Clinical Research Unit, Ho Chi Minh City, Viet Nam; 3 Cambridge Institute of Therapeutic Immunology & Infectious Disease (CITIID) Department of Medicine, University of Cambridge, Cambridge, UK

**Keywords:** Viral infection, Next-generation sequencing

## Abstract

There is still limited information on the diversity of viruses co-circulating in humans and animals. Here, we report data obtained from a large field collection of enteric samples taken from humans, pigs, rodents and other mammal hosts in Vietnam between 2012 and 2016. Each of 2100 stool or rectal swab samples was subjected to virally-enriched agnostic metagenomic sequencing; the short read sequence data are accessible from the European Nucleotide Archive (ENA). We link the sequence data to metadata on host type and demography and geographic location, distinguishing hospital patients, members of a cohort identified as a high risk of zoonotic infections (e.g. abattoir workers, rat traders) and animals. These data are suitable for further studies of virus diversity and virus discovery in humans and animals from Vietnam and to identify viruses found in multiple hosts that are potentially zoonotic.

## Abstract (summary)

**Table:** 

Measurement(s)	viral metagenome
Technology Type(s)	DNA Sequencing
Factor Type(s)	related animal
Sample Characteristic - Organism	Homo sapiens • Rodentia • Chiroptera • Simiiformes • Sus • Carnivora • Capra hircus
Sample Characteristic - Environment	fecal environment
Sample Characteristic - Location	Viet Nam

Machine-accessible metadata file describing the reported data: 10.6084/m9.figshare.9889709

## Background & Summary

Viruses of zoonotic origin are a significant public health concern worldwide having given rise to a number of high profile health emergencies in recent years, including SARS and MERS coronaviruses, H5N1 influenza A and ebolavirus^1^. However, detailed information on the epidemiology and evolution of viruses co-circulating in human and animal populations in the absence of significant outbreaks or epidemics is generally lacking. To help overcome this deficiency, the Vietnam Initiative on Zoonotic InfectIONS (VIZIONS) project ran between 2011 and 2016 with the overall aim of improving understanding of the epidemiology and evolution of zoonotic virus infections (particularly emerging infections) in Vietnam in south-east Asia.

VIZIONS was a Wellcome Trust-funded collaboration between the Oxford University Clinical Research Unit (OUCRU) in Ho Chi Minh City, Vietnam, the Sanger Institute and the University of Edinburgh in the UK, and Metabiota Inc. in the US, working closely with seven Vietnam hospitals, Hanoi Medical University, and Vietnam regional medical centres and sub-departments for animal health^1^.

VIZIONS supported studies in hospitals, communities and animal populations throughout Vietnam covering mainly enteric, respiratory, CNS infections and jaundice and generating data on clinical symptoms and outcomes, diagnostics, epidemiology and risk factors, virus identification, and virus genome sequences^2,3^. The data reported here comprise metagenomic sequence data and linked metadata for 2100 enteric samples from humans and animals and come from two major components of the VIZIONS project, one hospital-based and one community-based.

The first component was a large hospital-based surveillance study: a retrospective case series of hospitalised patients presenting with enteric disease believed to result from an infection^3^. The main aims of the study were to obtain high resolution data on all viruses present in these patients; to identify viruses of possible zoonotic origin causing severe disease (resulting in hospitalisation); and to estimate the disease burden due to viruses of zoonotic origin. This component contributed two additional sub-studies: a small study of only patients who tested positive for rotavirus A infection using a PCR test; and another small study of patients with undiagnosed enteric disease at four hospital sites.

The second component was a high-risk sentinel cohort: a prospective longitudinal cohort of people who have frequent occupational contact with animals and are thus perceived to be at high risk of developing zoonotic infections^2^. This component contributed two sub-studies: the high-risk sentinel cohort itself; and a parallel study of non-human mammals with which the human subjects were in close contact. The main aims of the study were to characterise the diversity of viruses circulating in high-risk human populations and in the animals with which they were in contact (including those that were asymptomatic or caused only mild illness that would not be picked up in a hospital-based study), estimate the incidence of virus infections in the human cohort, and identify possible instances of cross-species transmission. Subjects were selected based on high levels of exposure to animals (mainly on farms, in wet markets or slaughterhouses) and were sampled routinely at regular intervals over a three-year period and during episodes of diarrhoea.

A metagenomic sequencing protocol using random primers and a reverse transcription step was used to identify viruses in faecal samples and rectal swabs from these studies. This method was considered to be less biased towards common pathogens and to yield more information than a battery of diagnostic PCRs targeting specific, known pathogens. Metagenomic sequence data would allow in-depth characterisation of virus diversity in the study populations. Subsequent phylogenetic analysis would provide insights into the evolutionary and epidemiological dynamics of the identified viruses, including their spatial and temporal spread through human and animal populations and the inference of any zoonotic transmission events.

**Fig. 1:**
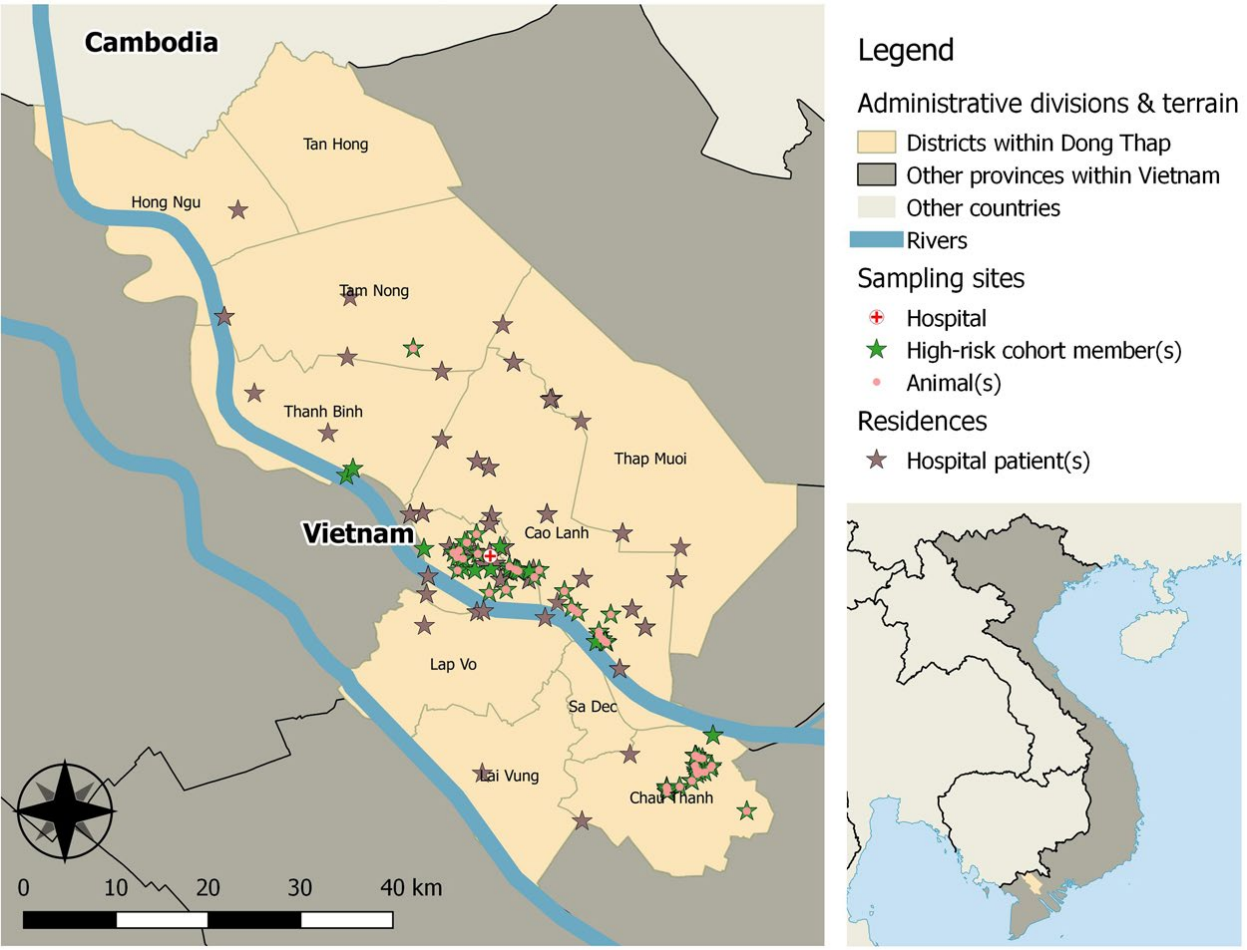
Map indicating approximate geographical locations of subjects and sampling points within Dong Thap province. Inset shows position of Dong Thap province within Vietnam.

The great majority of the data reported here were obtained from Dong Thap, a province in the south of Vietnam that is regarded as a potential hot-spot for the emergence of novel zoonotic viruses. Dong Thap province is in the Mekong Delta region (Fig. 1). Due to its mostly flat terrain, the Mekong Delta is one of the most important agricultural centres in Vietnam, and it has high human and livestock population densities. Dong Thap has an area of 3,379 km^2^ and is inhabited by just under 1.7 million people, approximately 4.7 million ducks, chickens and other poultry, 233,000 pigs, and 26,000 cattle and buffalo^3^. Additionally, there is a vibrant rat trade, with many tonnes of live rats sold for human consumption each year^2^. In addition, a small number of samples are included from other sites in Vietnam (see below).

Overall, sequence data and metadata are available for 2100 samples collected in Vietnam between November 2012 and May 2016. The numbers of samples from each of the five sub-studies are shown in Table 1.

**Table 1:** Origin of samples: overview.

Study population	Province(s)	No. samples	Sampling dates
Hospital patients – enteric disease study	Dong Thap	602	Nov 2012–May 2015
Hospital patients – rotavirus study	Dong Thap	49	Mar 2014–May 2016
Hospital patients – DUO study	Dak Lak, Dong Thap, Khanh Hoa, Thua Thien Hue	56	Nov 2012–Jun 2014
High-risk cohort	Dong Thap	551	Mar 2013–May 2016
Non-human animals	Dong Thap	842	Mar 2013–Feb 2015

**Fig. 2:**
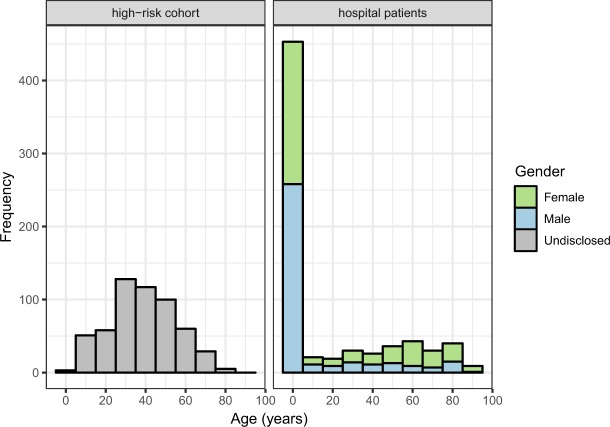
Age and gender distribution of participants in the high-risk and hospital based study. Age (years) and genders (male (blue), female (green)) were recorded for each participant in the hospital based study on the day of sampling. The age of participants in the high-risk cohort was recoded on the day of sampling. The genders of participants in the high-risk cohort were not disclosed.

1258 samples were obtained from human subjects (Fig. 2). 707 samples were collected from patients admitted to hospital with symptoms of enteric disease. The great majority of these (95%) were from Dong Thap General Hospital (Table 1). 551 samples were obtained from a high-risk sentinel cohort in Dong Thap province, most of whom were sampled on multiple occasions. These subjects are divided into four risk categories (Table 2). 842 samples were obtained from a variety of non-human animals in Dong Thap province in parallel with sampling the high-risk cohort (Table 3). Information on host species, sampling date, location and other attributes (see below) is available for all samples.

**Table 2:** Samples from high-risk cohort by risk category and sample type.

Risk category	Sites	Baseline (2013)		Clinical episodes		End (2016)		Total samples
		No. swabs	No. subjects	No. swabs	No. subjects.	No. stools	No. subjects	
Farm households	61	214	214	24	22	166	166	404
Animal health workers	3	30	30	7	6	23	23	60
Abattoir workers (pig slaughterers)	1	15	15	18	10	14	13	47
Poultry slaughterers	2	18	18	1	1	15	15	34
Rat traders	3	4	4	0	0	2	2	6
Total		281	281	50	39	220	219	551

**Table 3:** Non-human samples by host category and species.

Host category	Species	No. samples
Rat	*Rattus argentiventer*	279
	*Rattus losea*	20
	*Bandicota indica*	8
	*Rattus tanezumi*	8
Swine	*Sus domesticus*	278
	*Sus scrofa* (farmed wild boar)	7
Bat	*Scotophilus kuhlii*	173
	Unknown	6
Dog	*Canis familiaris*	45
Cat	*Felis catus*	13
Monkey	*Macaca arctoides*	1
	*Macaca leonina*	1
	Unknown	1
Goat	*Capra hircus*	2

**Fig. 3:**
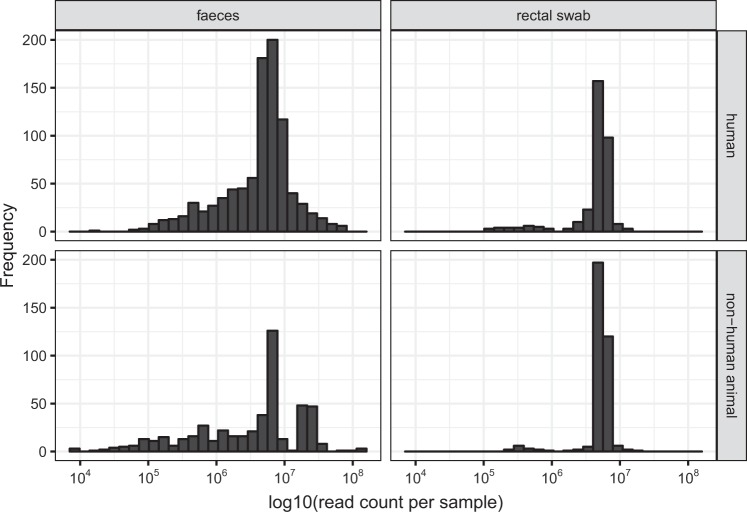
Distribution of log_10_(read count per sample) grouped by sample collection method and host type. For each sample, the total number of reads produced by metagenomic sequencing were calculated. Samples were split into four categories based on sample collection method (faecal sample or rectal swab) and host type (human or non-human animal).

Metagenomic sequencing yielded from 3670 to 59.5 M read pairs (Illlumina 2 × 250 nt reads) per sample, with a mean of 3.38 M, a median of 2.66 M and a mode of 3.1 M. 67.7% of samples were from stool and 32.3% were from rectal swabs. Stool samples had higher modal read counts (Fig. 3).

The sequence data and linked metadata have been used in a series of published research papers on viruses in Vietnam:

- Complete genome characterization of two wild-type measles viruses from Vietnamese infants^4^;
- Genome sequences of a novel Vietnamese bat bunyavirus^5^;
- Whole-genome deep sequencing of rotaviruses from human and porcine stool samples with evidence of a putative zoonotic infection^6^;
- Characterization of Posa and Posa-like virus genomes in faecal samples from humans, pigs, rats, and bats collected^7^;
- Genetic diversity and cross-species transmission of kobuviruses in Vietnam^8^;
- Virus diversity in enteric bat samples from Vietnam^9^;
- Identification of coronavirus genomes from bats and rodents^10^.

We anticipate that the data may be used for further studies of virus diversity and virus discovery in humans and animals from Vietnam and to identify viruses found in both kinds of host that are potentially zoonotic.

## Methods

Methodological summaries are provided below for each of the 5 sub-studies listed in Table 1. These are followed by a summary of the metagenomic sequencing methodology – this was common to all 5 sub-studies.

### Hospital patients – enteric disease study

#### Study design and recruitment

This study took place in Dong Thap General Hospital between November 2012 and May 2015. The study method are described in full elsewhere^1^. All hospital admissions during the study period with suspected enteric disease were considered for recruitment and up to six patients per day were enrolled into the study.

#### Patient inclusion and exclusion criteria

Patients were included in the study if they satisfied the following criteria: a clinical diagnosis of acute diarrhoeal disease (defined as a minimum of three loose stools within 24 hours, or one bloody loose stool) believed to be caused by an infection; provision of written informed consent; and requiring admission to the hospital as decided by the attending physician. Patients were excluded from the study if they had been hospitalised with enteric disease within the previous six months, if they had previously been enrolled to the study, if the diarrhoea was deemed likely to be due to prior antibiotic treatment, or if they had unrelated medical complications.

#### Data collection

Upon enrolment, study staff collected a stool sample (on the day of admission, before any antimicrobial treatment) and a variety of demographic and other epidemiologically relevant information. The patient’s residential address was recorded and mapped with GPS software. Stool samples were subjected to a range of diagnostic tests at the microbiology departments of the participating hospitals and at the central study laboratory at OUCRU: these data are incomplete and are not reported here. All 602 samples collected during this study were sent for metagenomic sequencing (see below).

### Hospital patients – rotavirus study

Faecal samples from a subset of hospital patients admitted to Dong Thap General Hospital with enteric disease tested positive by PCR test for the presence of rotavirus A (full details are provided elsewhere^6^). Between March 2014 and May 2016, 49 such patients were purposefully sampled in order to provide rotavirus positive samples as a partial validation of the metagenomic sequencing. These patients were additional to the routine sample collection from enteric disease patients described above. In all other respects these samples were treated in an identical manner to those from the enteric disease study.

### Hospital patients – disease of unknown origin (DUO) study

Between November 2012 and June 2014, stool samples from 56 hospital patients with an enteric disease of unknown origin (DUO) were sent for metagenomic sequencing. The samples were collected from Dak Lak Provincial Hospital (23), Khanh Hoa Provincial Hospital (8), Hue Provincial Hospital (8) and Dong Thap General Hospital (19). Inclusion and exclusion criteria were as for the main enteric disease study, with the additional criterion that no pathogen was identified by diagnostic screening at the hospital. In all other respects these samples were treated in an identical manner to those from the enteric disease study at Dong Thap General Hospital.

### High-risk sentinel cohort study

#### Study design and recruitment

This study took place in Dong Thap Province between March 2013 and May 2016. The study design, methods and baseline characteristics of the cohort are described in detail elsewhere^11,12^. Four categories of subjects with residential or occupational exposure to animals were recruited into the cohort:

- Animal farmers and their relatives, representing people with typical residential exposures to a variety of livestock species. This included keepers of both domestic and exotic animals, but excluded keepers of cold blooded animals. Poultry, pig and cattle farmers were originally selected at random from the animal farm census. Exotic animal farmers were actively sought as a group of special interest with the aim of maximising diversity of animals sampled. Up to four family members, including children, were enrolled from 61 farms.
- Pig and poultry slaughterers, representing people with more intense occupational exposures (particularly to blood). These were recruited from the three major abattoirs and slaughter points in Dong Thap Province.
- Animal health workers, representing people with intense occupational exposures to sick animals. These were recruited through the Dong Thap Sub-Department of Animal Health and District Veterinary Stations.
- Rat traders and sellers from three marketplaces. These were actively sought as a group of special interest.

#### Inclusion and exclusion criteria

Potential participants were included in the cohort study if they were long-term members of one of the above four risk groups; gave informed consent to sampling at regular intervals for three years; and resided within 40 km of Dong Thap General Hospital for clinical presentation in the event of illness.

#### Data collection

Included individuals were followed up for three years. Cohort members were requested to provide a rectal swab at enrolment, yearly thereafter, and when they reported an episode of enteric illness. In the third year of the study, stool samples were collected instead of rectal swabs, as it had become apparent that human rectal swabs generally did not have sufficient concentrations of viral nucleic acid for reliable metagenomic sequencing. Detailed demographic, socio-economic and behavioural data relating to food preparation and consumption were collected from each participant; these data are not reported here. A total of 551 samples were sent for metagenomic sequencing. These comprised:

- rectal swabs taken during baseline data collection (March–October 2013);
- rectal swabs from episodes of enteric illness (April–September 2013);
- faecal samples from the final data collection period (March–May 2016).

### Non-human animal study

The high-risk cohort study also included the sampling of associated mammals and birds. This part of the study is described in detail elsewhere^10^. A subset of 842 these samples was selected for metagenomic sequencing. This subset comprised all available rodent and bat samples, and a representative selection from other mammals (Table 3). Bat and monkey species were not recorded during sampling, therefore this was predicted by querying the raw reads from bat and monkey samples against mitochondrial DNA databases containing Chiroptera (NCBI:txid9397, February 2017) and Simiiformes (excluding *Homo sapiens*) (NCBI:txid314293, June 2018) sequences respectively. This was performed using Blastn (e value < 1e-50) and selecting the species with the highest frequency of hits.

### Viral metagenomic sequencing

A total of 2100 samples obtained from the 5 sub-studies were sent for metagenomic sequencing at the Sanger Institute, UK. Samples were stored at −80 °C after collection and maintained at this temperature until sample processing (up to a maximum of 12 months). Samples were processed according to a modified Virus Discovery by cDNA Amplified Fragment Length Polymorphism (VIDISCA) method^13–15^. This method enriches samples for viral genetic material, which is otherwise difficult to sequence as it is naturally present at much lower concentrations than host and bacterial nucleic acid. It then converts genetic material from both DNA and RNA viruses to dsDNA to allow for metagenomic library preparation, using a process that is sequence-independent and reduces contamination by host ribosomal RNA.

Detailed procedures were as follows. Samples were suspended in an equal volume of phosphate-buffered saline, and 110 µl were used for nucleic acid extractions. First, viral nucleic acid was enriched for by the removal of cellular debris, mitochondria and bacteria with a centrifugation step (10 minutes at 10,000 × g), and subsequent digestion of any residual DNA with DNase (20 units TURBO DNase, Ambion) - viral nucleic acid was presumed to be protected from this treatment by encapsidation within virions. Protected viral nucleic acid was then extracted using the Boom extraction method^16^. Viral RNA was converted to a cDNA intermediate using reverse transcriptase (Superscript II, Invitrogen) and a mixture of hexamer primers, designed to avoid binding to ribosomal RNA^17^. Subsequently, cDNA and viral ssDNA were subjected to second strand synthesis with 5 units Klenow fragment (3′-5′ exonuclease defective, New England Biolabs), and the resulting dsDNA was purified by phenol/chloroform extraction and ethanol precipitation.

#### Library preparation and sequencing

Isolated viral dsDNA was used to prepare metagenomic sequence libraries for deep sequencing, as previously described^6^. Library preparation followed standard Illumina protocols, with up to 96 samples being prepared at a time for multiplex sequencing. Nucleic acids were sheared to 400–500 nucleotides (nt) in length. Distinct 8 nt indexing barcodes were added to each sample’s nucleic acid before pooling. Each pool was sequenced divided over two lanes. Sequencing was done on Illumina HiSeq. 2500 machines, yielding up to several million 250 nt paired-end reads per sample. Adapters were trimmed off with Biobambam2^18^. For ethical reasons, human-derived sequences were identified by aligning reads to the GRCh38 reference genome (without Epstein-Barr virus) with BWA-backtrack^19^, and then removed. The resulting sequence data were deposited in the European Nucleotide Archive (ENA) in two batches, the first by Wellcome Sanger Institute as study PRJEB6505^20^ and the second by the University of Edinburgh as study PRJEB26687^21^. These sequencing studies are linked by umbrella study PRJEB27881^22^, where sequence data for each sample is available for download in fastq or cram format.

### Ethics

Informed consent was obtained from all human subjects. All sampling and testing procedures were carried out in accordance with guidelines that had previously been reviewed and approved by the relevant institutional ethics committees for human and veterinary medicine. This includes the Ethics Board of Dong Thap Hospital and the review board of sub-Department of Animal Health (for work carried out in Dong Thap); the Ethics Board Hospital of Tropical Diseases and the review board of the sub-Department of Animal Health (for work carried out in Dong Thap province) and the Hanoi Medical University and the review board of the sub-Department of Animal Health in Ha Noi (for work carried out in Ha Noi). In addition, all methods were approved by the Oxford Tropical Research Ethics Committee (OxTREC) (No. 157-12) in the United Kingdom.

## Data Records

The sample metadata are available in xslx format via Edinburgh DataShare^23^. The metagenomic sequence data are freely available through the ENA via studies PRJEB6505^20^ and PRJEB26687^21^ (linked under study PRJEB27881^22^). Data linkage is via ENA sample accession in Column T of the metadata file.

The metadata file contains the following information:

**Column A (Batch)** – Processing group identifier: samples with the same identifier were processed in the laboratory at approximately the same time. This may be useful information for users wanting to take batch effects into account.

**Column B (Run)** – Illumina HiSeq run identifier: samples with the same identifier were multiplexed and sequenced together at the Sanger Institute. This may be useful information for users wanting to take batch effects into account.

**Column C (Sample ID)** – Unique identifier for each sample and its corresponding sequence data.

**Column D (Subject ID)** – Identifier for each individual study subject (hospital patient, cohort member or animal). Study subjects may have had multiple samples taken, hence this identifier is not unique to a sample. It consists of a study code (04VZ for the hospital study, 05VZ for the cohort study), a location code (10 for Dong Thap hospital, or 75-nn for cohort study sites), and an individual identifier.

**Column E (Study component)** – VIZIONS study component under which the sample was collected.

**Column F (Sampling date)** – Sample collection date in format YYYY-MM-DD.

**Column G (Sampling occasion)** – Occasion on which the sample was collected. For patients in the hospital study, this is “hospitalisation (enteric disease)”. For high-risk cohort members, this is “study baseline”, “clinical episode”, or “study end”. For bats and rats, this is “routine sampling”. For other (farm) animals, this is “study baseline”.

**Column H (Host category)** – Host category of the study subject: “human”, “swine”, “cat”, “dog”, “goat”, “monkey”, “rat”, or “bat”.

**Column I (Host species)** – Species of the study subject (scientific name), if known.

**Column J (Risk category)** – Risk category of the high-risk cohort member: “abattoir worker (pig slaughterer)”, “animal health worker”, “farm household”, “rat trader”, “poultry slaughterer”.

**Column K (Age of host)** – Age of the study subject at time of sampling, in years.

**Column L (Sex/gender of host)** – Sex or gender of the study subject.

**Column M (Sample type)** – Sample type: “faeces” or “rectal swab”.

**Column N (Province)** – Province of the subject’s residence (for patients in the hospital study) or of the sampling site (for cohort members and animals).

**Column O (District)** – District of the subject’s residence (for patients in the hospital study) or of the sampling site (for cohort members and animals).

**Column P (Latitude)** – Latitude (degrees North) of the subject’s residence (for patients in the hospital study) or of the sampling site (for cohort members and animals). For samples collected in the community, latitude is reported to only 2 decimal places to preserve subjects’ anonymity.

**Column Q (Longitude)** – Longitude (degrees East) of the subject’s residence (for patients in the hospital study) or of the sampling site (for cohort members and animals). For samples collected in the community, longitude is reported to only 2 decimal places to preserve subjects’ anonymity.

**Column R (Study accession)** – ENA accession number for the study.

**Column S (Secondary study accession)** – ENA secondary accession number for the study.

**Column T (Sample accession)** – ENA accession number for the sample.

**Column U (Secondary sample accession)** – ENA secondary accession number for the sample.

**Column V (Read count)** - Number of raw reads produced upon sequencing the sample.

## Technical Validation

**Fig. 4:**
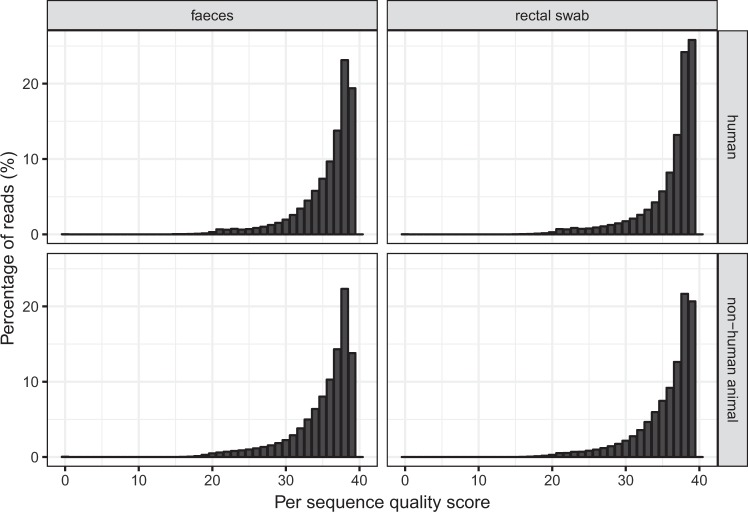
Average read quality grouped by sample collection method and host type. The average read quality for each read was calculated using FastQC. Reads were split into four categories based on collection method (faecal sample or rectal swab) and host type (human or non-human animal) of the sample they belong to.

RNA was quantified prior to sequencing using the Qubit RNA HS Assay Kit. Sequence data had adapters trimmed off and human-derived reads filtered out (as described in the Methods section). The average read quality (based on *Phred* score^24,25^) was determined for all samples using FastQC (www.bioinformatics.babraham.ac.uk/projects/fastqc/) (Fig. 4). Of the 14.4 billion reads generated, 99.59% had an average quality score of 20 or above. Users of the sequence data are responsible for performing their own quality checks.

Metadata were curated manually and all entries cross-checked by a data manager.

## Usage Notes

The metadata associated with the sequence data is located on Edinburgh DataVault^23^. As the VIZIONS metadata include anonymised information on human subjects it cannot be made openly available. Researchers are required formally to request access to the database by contacting the Corresponding Author. Researchers will be required to complete a Data Release Request form that covers the purpose of the study, funding, ethical approvals, publication and intellectual property (Supplementary File 1).

## Supplementary information

Supplementary File 1
