# Supplementary material for: Sample descriptors linked to metagenomic sequencing data from human and animal enteric samples from Vietnam
Source: Sci Data. 2019 Oct 15;6:202. doi: 10.1038/s41597-019-0215-2 (PMC6794271; doi:10.1038/s41597-019-0215-2)
Supplement: Supplementary file 1 [file 41597_2019_215_MOESM1_ESM.docx]

**DATA RELEASE REQUEST FORM FOR VIZIONS STUDIES**

| **Project Title** |  |
| --- | --- |
| **Lead Applicant** |  |
| **Application Date** | dd-mmm-yyyy |
| **Application Version** | X.XX |
| **VIZIONS data set requested** |  |
| **Project Code** |  |

**Notes**

This application form provides the basis for review of the proposed project and the development of the necessary agreements. Further supporting documents will be included

**1. Lead Applicant**

| **QUESTION** | **ANSWER** |
| --- | --- |
| **Lead Applicant Name** |  |
| **Lead Applicant Institution** |  |
| **Lead Applicant Address** |  |
| **Lead Applicant Telephone** |  |
| **Lead Applicant Email** |  |
| **List of Co-Applicants**  Include institutions |  |
| **Applicant Qualifications**  How is the team qualified to process and analyse the requested information, including analytic expertise? |  |

# 2. Project Summary and Supporting Information

This section should cover no more than two sides of A4

| **QUESTION** | **ANSWER** | | | |  |
| --- | --- | --- | --- | --- | --- |
| **2.1 Project Title** |  | | | |  |
| **2.2 Source of request**  Mark one |  | Internal to VIZIONS | | |  |
|  |  | External | | |  |
| **2.3 Motivation of request**  Mark all that apply |  | Replication of key analysis | | |  |
|  |  | Re-analysis with alternative methods | | |  |
|  |  | Meta-analysis or combination with other study data | | |  |
|  |  | Clinical | | |  |
|  |  | Basic science | | |  |
|  |  | Methodology | | |  |
|  |  | Other, specify: | | |  |
|  |  | | | |  |
| **2.4 Background**  What is the setting for the project?(Maximum 250 words) |  | | | |  |
| **2.5 Rationale***  What is the reason for undertaking the project? (Maximum 250 words) |  | | | |  |
| **2.6 Defined objectives**  What are the expected outputs or deliverables? (Max 150 words) |  | | | |  |
| **2.7 How is the project funded?**  How will the project be funded? Has this funding already been secured? |  | | | |  |
| **2.8 What other approvals are required for this project?**  Give details of ethics and regulatory submissions, and peer review processes. Include reference numbers and dates if already awarded. |  | | | | |
| **2.9 Provide relevant documents to support the application**  These should be included as separate documents with this application form | **Document** | | **Status** | | |
|  | Protocol | |  | Attached | |
|  |  |  |  | Not attached | |
|  |  |  |  | Not applicable | |
|  | Statistical Analysis  Plan | |  | Attached | |
|  |  |  |  | Not attached | |
|  |  |  |  | Not applicable | |
|  | Grant application | |  | Attached | |
|  |  |  |  | Not attached | |
|  |  |  |  | Not applicable | |
|  | Other documents  eg ethics committee approval | |  | Attached: specify below | |
|  |  |  |  | | |
|  |  |  |  | Not attached | |
|  |  |  |  | Not applicable | |

* Keeping in mind the potential impact and benefit to the larger scientific community

# 3. Study Data and Requirements

| **QUESTION** | **ANSWER** |
| --- | --- |
| **3.1 Why are data from this study requested for this project?** |  |
| **3.2 Why are they requested now?** |  |
| **3.3 How does the proposal fit with the study’s consent process?**  Provide clarification and justification, preferable including a copy of the PIS highlighting the relevant parts; If not, give details of the proposed process |  |
| **3.4 Is summary data requested or individual participant data** |  |
| **3.5 What data are required?**  Broad description of variables, with detailed list as further document, if possible. Which visits are needed? All participants or a subset? |  |
| **3.6 What are the timelines for the project?**  Include when is data required by and when the project will report. |  |
| **3.7 Which version of the data are required?**  Does this relate to the data used for a particular analysis or publication, or to the current data, or to a future dataset? |  |
| **3.8 What efforts and activities**  **are requested of VIZIONS?**  eg Access to data, provision of background information |  |
| **3.9 What processes are in place to support the activities by VIZIONS?**  eg financial support to cover staff time |  |
| **3.10 Where will data be sent and what are the specific regulatory issues in such a transfer?** |  |

# 4. Publication Policy and Intellectual Property Rights

| **QUESTION** | **ANSWER** | |
| --- | --- | --- |
| **4.1 Publication policy**  How and where do you plan to present and publish the results of this project? |  | |
| **4.2 Open Access publishing**  Data should be published in an Open Access format and paid for by the Applicant |  | Following Gold open access publishing policy |
|  |  | Following green open access publishing policy |
|  |  | No open access publishing: explain below |
|  |  | |
| **4.3 Intellectual property rights**  The data remain under VIZIONS custodianship. Specify what will be the proposed intellectual property rights over the outputs and deliverables. IPR will be finalized in a formal agreement. |  | |

# 5. Standard Conditions

These are the standard conditions under which data or samples collected or pledged as a part of a VIZIONS OUCRU-led study, and stored in the VIZIONS database under University of Edinburgh guardianship, can be shared with external groups for research purposes. A specific agreement would be developed between the appropriate bodies if approval is granted for data and/or samples to be released.

1. The research project described must conform to relevant ethics and research governance requirements and the Applicant must provide evidence of this before data or samples are released.
2. Data will only be released once approval has been obtained from the appropriate parties. A decision on approval will be based on a review of the detailed description of the project and the feasibility of the data extraction and/or data transfer and/or sample provision.
3. The data transferred are confidential, must be stored in a secure location, must not serve for any other purpose than those specified in the application for which approval for release is given and must not be discussed outside of the working group for the project named in this document. Any samples provided should only be used for the purposes specified in this document.
4. The Applicant must keep the link person at VIZIONS informed of the process of the project and must provide any draft publication for review before it is used in any type of public presentation or submitted for publication. All outputs should acknowledge VIZIONS as the source of the data. A reprint of the resulting publication should be provided to VIZIONS as soon as available.
5. The Applicant will be expected to follow and pay for Open Access Publication.
6. Upon completion of the project or publication of the results, all copies of the data held must be archived securely following University of Edinburgh guidelines or destroyed. No data can be shared on without discussion and consent with VIZIONS.
7. The University of Edinburgh is the custodian of all the data and VIZIONS holds the Intellectual Property Rights over the data and subsequent outputs unless otherwise clarified in a separate agreement.
8. This document considers the principles of data release if permission is granted. A formal agreement must be drawn up and signed by the legal representatives of the parties involved.
9. The recipients of data should regularly update VIZIONS on the progress of their project until the point of completion.

**6. Signature**

The answers in this document are true and accurate to the best of my knowledge. I have read and understood the conditions above.

**Applicant Name:**

**Applicant signature:**

**Date:**
